# Circular RNA COL1A1 promotes Warburg effect and tumor growth in nasopharyngeal carcinoma

**DOI:** 10.1007/s12672-024-00941-1

**Published:** 2024-04-15

**Authors:** ZeJun Zhou, Fang Xu, Tao Zhang

**Affiliations:** 1 Department of Otolaryngology, The First Affiliated Hospital of Jinan University, No. 613 West Huangpu Avenue, Tianhe District, Guangzhou, 510630 Guangdong China; 2 Health Management Center, The First Affiliated Hospital of Jinan University, Guangzhou, 510630 Guangdong China

**Keywords:** Nasopharyngeal carcinoma, circCOL1A1, miR-370-5p, PTMA, Warburg effect

## Abstract

**Objective:**

Circular RNAs (circRNAs), pivotal in the pathogenesis and progression of nasopharyngeal carcinoma (NPC), remain a significant point of investigation for potential therapeutic interventions. Our research was driven by the objective to decipher the roles and underlying mechanisms of hsa_circ_0044569 (circCOL1A1) in governing the malignant phenotypes and the Warburg effect in NPC.

**Methods:**

We systematically collected samples from NPC tissues and normal nasopharyngeal epithelial counterparts. The expression levels of circCOL1A1, microRNA-370-5p (miR-370-5p), and prothymosin alpha (PTMA) were quantitatively determined using quantitative polymerase chain reaction (qPCR) and Western blotting. Transfections in NPC cell lines were conducted using small interfering RNAs (siRNAs) or vectors carrying the pcDNA 3.1 construct for overexpression studies. We interrogated the circCOL1A1/miR-370-5p/PTMA axis's role in cellular functions through a series of assays: 3-(4,5-dimethylthiazol-2-yl)-2,5-diphenyltetrazolium bromide for cell viability, colony formation for growth, Transwell assays for migration and invasion, and Western blotting for protein expression profiling. To elucidate the molecular interactions, we employed luciferase reporter assays and RNA immunoprecipitation techniques.

**Results:**

Our investigations revealed that circCOL1A1 was a stable circRNA, highly expressed in both NPC tissues and derived cell lines. A correlation analysis with clinical pathological features demonstrated a significant association between circCOL1A1 expression, lymph node metastasis, and the tumor node metastasis staging system of NPC. Functionally, silencing circCOL1A1 led to substantial suppression of cell proliferation, migration, invasion, and metabolic alterations characteristic of the Warburg effect in NPC cells. At the molecular level, circCOL1A1 appeared to modulate PTMA expression by acting as a competitive endogenous RNA or 'sponge' for miR-370-5p, which in turn promoted the malignant characteristics of NPC cells.

**Conclusion:**

To conclude, our findings delineate that circCOL1A1 exerts its oncogenic influence in NPC through the modulation of the miR-370-5p/PTMA signaling axis.

**Supplementary Information:**

The online version contains supplementary material available at 10.1007/s12672-024-00941-1.

## Introduction

Nasopharyngeal carcinoma (NPC) is a squamous cell carcinoma of the head and neck with high aggressiveness [1]. Diagnosis of advanced NPC happens in more than 70% of tumor cases due to the insidious symptoms of NPC, tumor aggressiveness, and poor patient awareness [2]. Under the comprehensive treatment of radiotherapy, the 5-year survival rate of NPC has reached about 85–90% [3, 4]. However, about 8–10% of patients still experience disease recurrence or distant metastasis, which is the main reason for treatment failure in some patients [4]. Therefore, the identification of novel and sensitive biomarkers is needed to develop new therapeutic strategies.

Unlike normal differentiated cells, most cancer cells rely on aerobic glycolysis to obtain high energy to rapidly adapt to growth, a phenomenon known as the "Warburg effect" [5–7]. Therefore, a proposal that targeted blockade of Warburg effect has therapeutic prospects to control cancers [8, 9]. Aberrant glycolysis is closely associated with NPC proliferation and metastasis [10–12]. However, the mechanism for regulating metabolism at the post-transcriptional level is still insufficient, such as the role of circular RNAs (circRNAs) and microRNAs (miRNAs).

The study of circRNAs has become the frontier of biomedical research [13, 14]. CircRNAs with covalently closed loop structures are characterized by high stability and abundance [15, 16]. The competing endogenous RNA mechanism is recognized as the main regulatory mechanism of circRNAs in regulating biological processes [17]. Furthermore, many studies have revealed that circRNAs participate in malignant biological processes, including Warburg effect [18, 19]. Many circRNAs have been identified to be differentially expressed in NPC, and have the potential to regulate the malignant behavior of NPC [20], such as circular RNA Ran-binding protein 17 [21] and circ-itchy E3 ubiquitin protein ligase [22]. Our research group noticed that hsa_circ_0044569 (circCOL1A1) is a novel oncogenic circRNA in gastric cancer that is involved in the malignant behavior of cancer cells [23]. In the preliminary experiment, circCOL1A1 was found to be significantly up-regulated in NPC tissues by PCR analysis. This finding suggests that circCOL1A1 may play a role in the biological process of NPC. Given the limited understanding of the role of circCOL1A1 in NPC, our study aims to fill this gap.

The purpose of this study was to investigate the effect of circCOL1A1 on the malignant behavior of NPC cells and its mechanism. These results may provide a new perspective for Warburg effect in NPC progression, and more importantly, may also provide a new theoretical basis for targeted therapy of NPC.

## Materials and methods

### Clinical samples

NPC tissues (n = 42) and normal nasopharyngeal epithelial tissues (n = 16) were collected from The First Affiliated Hospital of Jinan University. All tissue samples were confirmed by pathological examination. Neither radiation, chemotherapy nor other treatments were received by patients. The resected samples were stored at − 80 °C until RNA or protein extraction. The ethics committee of The First Affiliated Hospital of Jinan University provided approval for the study and all patients provided written informed consent (Ethical approval number: 202108GZ2161). Table 1 shows clinical information on the subjects.

**Table 1:** Clinical characteristics of healthy subjects and NPC patients

Data item	Healthy subjects (n = 16)	NPC patients (n = 42)	P value
Average age (years)	45.94	43.02	0.127
Age standard deviation (years)	14.56	15.27	
Average weight (kg)	80.75	75.79	0.262
Weight standard deviation (kg)	16.02	14.50	
BMI average	24.94	26.22	0.324
BMI standard deviation	5.54	5.02	
Smoking history (yes) (cases)	6	21	0.576
Drinking history (yes) (cases)	6	24	0.296
Hypertension history (yes) (cases)	3	17	0.212

### Cell culture

Human NPC cell lines SNU46, SUNE1, CNE-1, HONE-1 and 6-10B, and human immortalized nasopharyngeal epithelial cell line (NP-69) were obtained from Cell Bank of the Chinese Academy of Sciences (Shanghai, China). Cells were grown in Roswell Park Memorial Institute-1640 medium (Gibco, USA), 10% fetal bovine serum (FBS, Gibco), and 1% streptomycin/penicillin (Invitrogen, Carlsbad, USA) at 37 °C under 5% CO_2_. All cells were validated by short tandem repeat assays and detected negative for mycoplasma contamination.

### Real-time reverse transcriptase-polymerase chain reaction (RT-qPCR)

Total RNA extracts were obtained using Trizol (Invitrogen). The complementary DNA (cDNA) synthesis for circRNA and mRNA was performed using the HiScript II first-strand cDNA synthesis kit (Vazyme, Nanjing, China). The cDNA synthesis for miRNA was carried out using the All-in-One miRNA first-strand cDNA synthesis kit (GeneCopoeia, Rockville, MD, USA). Gene expression was detected on a 7500 Real-Time PCR System (Applied Biosystems). GAPDH and U6 were internal controls. Gene expression was normalized by 2^−ΔΔCt^ method. All primers are listed in Table 2.

**Table 2:** RT-qPCR primer sequence

	Primer sequence (5'–3')
Has_circ_0044569	Forward: 5'-ACCCACCGACCAAGAAACC-3'
	Reverse: 5'-TTGTCGCAGACGCAGATC-3'
MiR-370-5p	Forward: 5'-ACACTCCAGCTGGGCAGGTCACGTCTCTGC-3'
	Reverse: 5'-TGGTGTCGTGGAGTCG-3'
PTMA	Forward: 5'-GAGGTAGACGAAGAAGAG-3'
	Reverse: 5'- GAAGTGGAGGGTGAATAG-3'
GAPDH	Forward: 5'-CACCCACTCCTCCACCTTTG-3'
	Reverse: 5'-CCACCACCCTGTTGCTGTAG-3'
U6	Forward: 5'-CTCGCTTCGGCAGCACA-3'
	Reverse: 5'-AACGCTTCACGAATTTGCGT-3'

### Subcellular isolation

circCOL1A1 localization was assessed using PARIS™ kit (Thermo Fisher Scientific). Cells were subjected to incubation in a lysis buffer followed by centrifugation at 12,000 × *g*. The resulting supernatant and nuclear precipitation were utilized for the extraction of RNA. Subsequently, RT-qPCR was conducted to evaluate the levels of circCOL1A1 in both the cytoplasmic and nuclear compartments of cells. 18S rRNA was chosen as the control for the cytoplasmic portion, while U6 served as the control for the nuclear portion.

### Actinomycin D and RNase R assays

To examine the resilience of circCOL1A1 within the cellular milieu of SUNE1 cells, we conducted a series of assays utilizing actinomycin D and RNase R treatments. For the actinomycin D assay, SUNE1 cells were cultivated in a nutrient-rich medium fortified with 2 μg/mL of actinomycin D (Sigma, MO, USA) to inhibit transcriptional activity. A parallel culture was maintained in a medium containing dimethyl sulfoxide (Sigma) to serve as the vehicle control. In parallel, for the assessment of RNA stability against enzymatic degradation, total RNA from SUNE1 cells was incubated with RNase R (3 U/μg, obtained from Geneseed, Guangzhou, China), an enzyme that selectively digests linear RNA, or treated with water that had been rendered inert with diethyl pyrocarbonate (Sigma) to ensure an RNase-free environment. The quantification of circCOL1A1 and the linear COL1A1 isoform was meticulously performed via RT-qPCR to evaluate the differential stability and expression post-treatment.

### Plasmid engineering

Small interfering RNA targeting circCOL1A1 and prothymosin alpha (PTMA) and negative control (NC) (si-circCOL1A1, si-PTMA, si-NC), overexpression plasmids targeting circCOL1A1 and PTMA, and NC (oe-circCOL1A1, oe-PTMA and oe-NC), miR-370-5p mimic/inhibitor, mimic/inhibitor NC were purchased from Gene Pharma (Shanghai, China). Vector and oligonucleotide were transfected into cells with Lipofectamine 3000 (Invitrogen). After 48 h, the transfection efficiency was assessed by RT-qPCR.

### 3-(4,5-Dimethylthiazol-2-yl)-2,5-diphenyltetrazolium bromide (MTT) method

A total of 5 × 10^3^ cells were introduced into 96-well plates and incubated for 24, 48, or 72 h. Following the removal of the supernatant, 100 μL MTT solution (0.5 mg/mL, Sigma) was added to each well and incubated for a duration of 4 h. Subsequently, the purple crystal was dissolved using dimethyl sulfoxide (150 μL, Sigma). Finally, the absorbance at 570 nm was measured using a microplate reader (Bio-Rad, Hercules, CA, USA).

### Colonies detection

A total of 5 × 10^3^ cells per pore were introduced onto a 6-well plate. Following a two-week incubation period, the cells were immobilized using 4% paraformaldehyde (Sigma) and subsequently subjected to staining with 0.5% crystal violet (Sigma). Enumeration of colonies was performed utilizing an inverted microscope (Nikon, Tokyo, Japan).

### Transwell assays

Transwell chambers with 8-μm pores (Corning, NY, USA) were utilized to determine cell invasion and migration. The chambers were coated or not coated with Matrigel (50 μL, BD Biosciences, NJ, USA) [24]. A total of 5 × 10^4^ cells suspended in serum-free medium were introduced into the upper chamber (upper chamber volume 200 μL). The lower chamber (lower chamber volume 600 μL) contained medium supplemented with 10% FBS. After 24 h, the cells were fixed with paraformaldehyde and stained with 0.1% crystal violet. The resulting samples were observed under a microscope at a magnification of 100 times (Olympus). Cell counts were determined using image analysis software.

### Glycolysis analysis

Cellular glucose consumption, lactic acid production and adenosine triphosphate (ATP) levels were measured as previously mentioned [25]. Glucose assay kits (Sigma), lactate colorimetric/fluorometric kits, and ATP assay kit (Thermo Fisher Scientific) were purchased to determine glucose consumption, lactate production, and ATP level, respectively.

### ATP/adenosine diphosphate (ADP) detection

ATP/ADP ratio was measured using the ApoSENSOR ADP/ATP Ratio Assay Kit (#K255-200, BioVision) [26]. The luminescence was quantified using a spectroscopic technique (Molecular Devices, Cal, US). A total of 1 × 10^4^ cells were introduced into the photometer and subsequently incubated with a nucleotide release buffer. Following this, 1 μL ATP-monitoring enzyme was added. The luminescence was recorded for a duration of 1 min (Data A) and 10 min (Data B). Subsequently, ADP convertase was introduced and the corresponding sample values were obtained (Data C). The ATP/ADP ratio was calculated as Data A divided by (Data C minus Data B).

### Nicotinamide adenine dinucleotide (NAD)^+^/Nicotinamide adenine dinucleotide (NADH) determination

NAD^+^/NADH ratio was tested using the EnzyChrom™ NAD^+^/NADH Ratio Assay Kit (E2ND-100, Bioassay Systems, CA, USA) [27]. Cells were suspended in NAD^+^ and NADH extraction buffers to conduct NAD^+^ and NADH assays. A total of 1 × 10^5^ cells were collected. The resulting homogenate was placed in a 1.5 ml Eppendorf tube, with 100 ml NAD extraction buffer added for NAD assay or 100 ml NADH extraction buffer added for NADH assay. Subsequently, 20 μl assay buffer was introduced to the extract, followed by the addition of 100 ml back extraction buffer to neutralize the extract. The mixture was then centrifuged, and the resulting supernatant was transferred to the working reagent. Optical density values at 565 nm were measured at both 0 and 15 min.

### Immunoblotting

Proteins present in cells and tissues were extracted utilizing the radioimmunoprecipitation lysis buffer (Beyotime). The quantification of protein concentration was accomplished using the bicinchoninic acid Protein Assay Kit (Beyotime). Subsequently, all cell lysates, each containing 40 μg protein, were subjected to SDS-PAGE and subsequently transferred onto polyvinylidene fluoride membranes through electrophoretic imprinting. The membrane was sealed with Tween-Tris buffered saline containing 5% skim milk at room temperature for a duration of 2 h. Subsequently, it was incubated with primary antibodies, namely GAPDH (2118, Cell Signaling Technology), PTMA (YN2871, Immunoway), hexokinase 2 (HK2; 22029-1-AP, Proteintech), and pyruvate kinases type M2 (PKM2; 4053, Cell Signaling Technology). Following this, the membrane containing protein bands was incubated with horseradish peroxidase-polymerized secondary antibody at room temperature for another 2 h. The imprints were visualized using the enhanced chemiluminescence detection system. The software FluorChem2.0 was employed to calculate the integral density values.

### Luciferase activity

The putative miR-370-5p target binding sequences and mutant sequences in circCOL1A1 and PTMA 3'untranslated region (UTR) were synthesized and cloned into the pmirGLO-promoter vector (Promega). Wild-type pmirGLO-circCOL1A1/PTMA (or mutant pmirGLO-circCOL1A1/PTMA) reporter plasmid was transfected with miR-370-5p mimic/inhibitor or mimic/inhibitor NC into cells via Lipofectamine 3000 (Invitrogen). The dual luciferase reporter gene assay system (Promega) measured firefly luciferase and renilla luciferase 48 h after transfection. The ratio of firefly to renilla luciferase activity is considered to be relative to luciferase activity.

### RNA immunoprecipitation (RIP) experiment

RIP experiments were performed according to the manufacturer (Magna RIP™, Millipore, MA, USA) (Supplementary File 1). Cells were lysed using RIP buffer to release RNA and proteins. RNase inhibitors and DNase I were added to avoid RNA degradation and DNA contamination. The specific antibody Ago2 antibody was added to the lysate and incubated overnight to form antibody-protein complexes. Subsequently, mouse IgG-coupled A/G magnetic beads were added to allow binding of the beads to the antibody complex. The complexes were harvested with proteinase K and the immunoprecipitated RNA was then isolated. RNA concentration measurements and RNA quality assessment were performed by spectrophotometer (NanoDrop, Thermo Scientific, MA, USA). To demonstrate the presence of binding targets, purified RNA was collected and tested by RT-qPCR.

### Data analysis

Data were obtained from at least three biological replicates and assessed by GraphPad Prism 9.0 software. Shown as mean ± standard deviation, the data were compared by one-way analysis of variance and Tukey's multiple comparison test or unpaired Student's t-test. Clinical association between NPC patients’ features and circCOL1A1 expression was analyzed by chi-square test. *P* < 0.05 was taken as a hallmark of significant difference.

## Results

### CircCOL1A1 is highly expressed in NPC

**Fig. 1:**
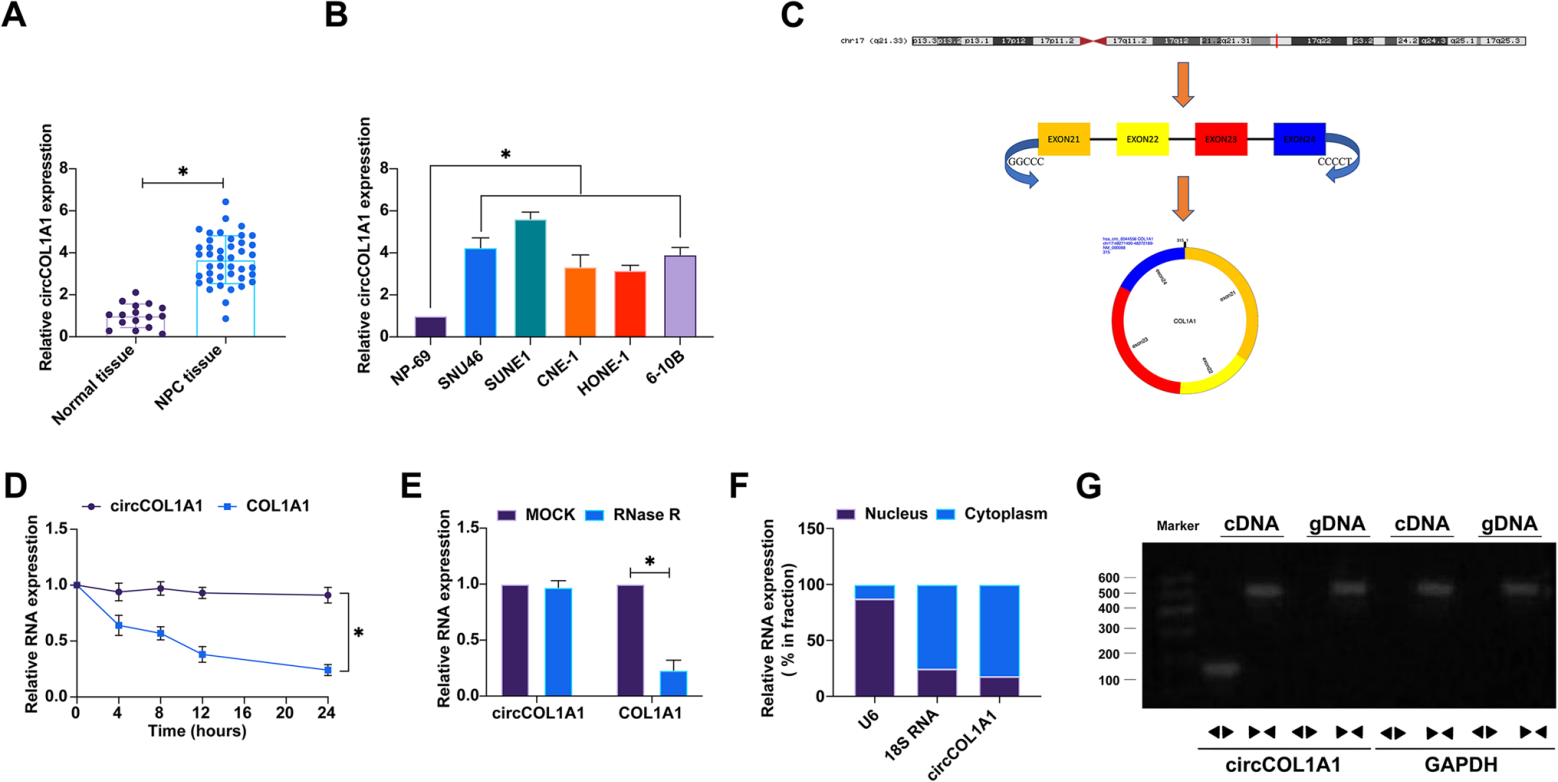
CircCOL1A1 is highly expressed in NPC. **A** RNA analysis of circCOL1A1 in NPC tissues and normal tissues; **B** RNA analysis of circCOL1A1 in NPC cell lines and normal cells; **C** circbase database to query the gene structure of circCOL1A1; **D**, **E** Actinomycin D and RNase R administration to determine the ring structure of circCOL1A1; **F** Subcellular separation to detect the expression site of circCOL1A1 in cells; data are expressed as mean ± standard deviation. *P < 0.05

To evaluate the differential expression of circCOL1A1 (hsa_circ_0044569) in nasopharyngeal carcinoma (NPC), our initial approach encompassed the quantitation of circCOL1A1 within NPC tissues and cell lines using RT-qPCR. The results demonstrated a pronounced upregulation of circCOL1A1 in NPC tissues in comparison to non-carcinomatous controls (Fig. 1A). Moreover, circCOL1A1 was consistently expressed at higher levels across a panel of NPC cell lines, namely SNU46, SUNE1, CNE-1, HONE-1, and 6-10B, when contrasted with the immortalized nasopharyngeal epithelial cell line NP-69 (Fig. 1B). Further investigation into the genomic architecture of circCOL1A1 via the circBase repository revealed that this 230 bp circular RNA species (chr17:48276916–48277308) is derived from exons 2 and 3 of the COL1A1 gene, located at the 17q21.33 locus (Fig. 1C). Validation of the circular topology of circCOL1A1 was affirmed by its resistance to RNase R digestion and actinomycin D treatment, which established a more stable entity in contrast to its linear mRNA analog (Fig. 1D, E). Linear COL1A1 mRNA was susceptible to degradation by RNase R, whilst circCOL1A1 remained intact. Furthermore, the existence of circCOL1A1 was corroborated by PCR, utilizing divergent and convergent primers for cDNA and gDNA, respectively (Fig. 1F). Subcellular localization studies through fractionation assays pinpointed the cytoplasmic predominance of circCOL1A1 expression (Fig. 1G). Stratification of NPC patients into cohorts with high and low circCOL1A1 expression, based on the median expression value, facilitated correlation analyses with clinicopathological parameters. These analyses disclosed a significant association of elevated circCOL1A1 expression with the incidence of lymph node metastasis and advanced tumor node metastasis (TNM) staging in NPC (Table 3). These integrative data underscore the heightened expression of circCOL1A1 in NPC and implicate its potential involvement in the molecular etiology of the disease.

**Table 3:** Correlation analysis of circCOL1A1 expression and clinicopathological characteristics of nasopharyngeal carcinoma patients

Characteristic	Cases	The expression of circCOL1A1		*P*
	n = 42	High(n = 21)	Low(n = 21)	
*Age (year)*				0.0601
≥ 60	33	19	14	
< 60	9	2	7	
*Gender*				0.1904
Male	14	5	9	
Female	28	16	12	
*Tumor size (cm)*				0.3523
≥ 5	19	11	8	
< 5	23	10	13	
*TNM stage*				0.0011*
I–II	28	9	19	
II–IV	14	12	2	
*Lymph node metastasis*				0.0038*
Negative	32	12	20	
Positive	10	9	1	

### Knockdown of circCOL1A1 inhibits malignant behaviors and Warburg effect in NPC

**Fig. 2:**
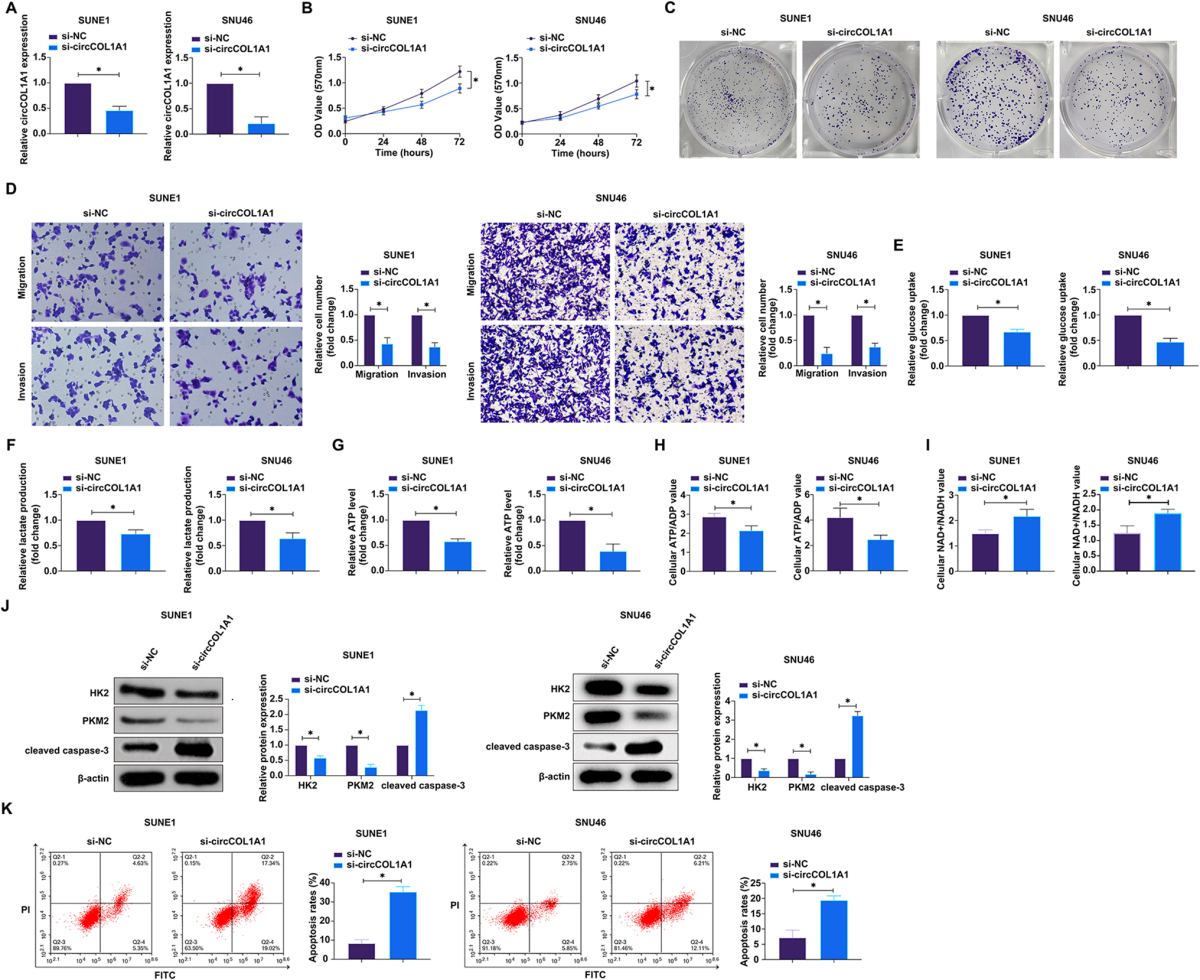
Silencing circCOL1A1 attenuates proliferation, invasion, migration, and the warburg effect in NPC. **A** circCOL1A1 expression was detected via RT-qPCR; **B** Cell proliferation was measured using the MTT assay; **C** Clonogenic ability was evaluated by plate colony formation assay; **D** Cell invasion and migration capabilities were quantified using Transwell assays; **E–I**: Metabolic assays were employed to determine glucose consumption, lactate production, ATP levels, ATP/ADP ratio, and NAD^+^/NADH ratio; **J** Western blot analysis was used to detect the expression of cleaved caspase-3, HK2 and PKM2 proteins; **K** Apoptosis rate was detected by flow cytometry. Data are presented as mean ± standard deviation (n = 3). * *P* < 0.05 indicates statistical significance. *NPC* nasopharyngeal carcinoma, *RT-qPCR* Real-time reverse transcriptase-polymerase chain reaction, *MTT* 3-(4,5-dimethylthiazol-2-yl)-2,5-diphenyltetrazolium bromide, *ATP/ADP ratio* adenosine triphosphate/adenosine diphosphate ratio, *NAD*^*+*^*/NADH* Nicotinamide adenine dinucleotide^+^/Nicotinamide adenine dinucleotide, *HK2* hexokinase 2, *PKM2* pyruvate kinases type M2

Subsequent investigations focused on the functional role of circCOL1A1 within the oncogenic landscape of NPC. Specific siRNA-mediated attenuation of circCOL1A1 was achieved, as evidenced by the marked reduction in its expression (Fig. 2A). This knockdown was functionally correlated with a decrement in cellular proliferation, as measured by MTT assays, and a significant reduction in clonogenic survival (Fig. 2B, C). In assessing the capability of NPC cells for invasive behavior post-circCOL1A1 silencing, transwell assays quantitatively confirmed that siRNA against circCOL1A1 significantly thwarted the invasive and migratory aptitudes of both SUNE1 and SNU46 cell lines (Fig. 2D). The metabolic repercussions of circCOL1A1 knockdown were evident in the perturbed glycolytic flux; there was a notable decrease in glucose uptake, lactate secretion, total intracellular ATP concentrations, and the ATP/ADP ratio, coupled with an elevation in the NAD + /NADH ratio within the circCOL1A1-depleted SUNE1 and SNU46 cells (Figs. 2E–I). To explore the potential mechanistic underpinnings of these metabolic alterations, we interrogated the expression of key glycolytic enzymes through western blot analysis. The data revealed that circCOL1A1 knockdown substantially diminished the expression levels of HK2 and PKM2 (Fig. 2J), which are pivotal regulators of the glycolytic pathway. Flow cytometry showed that knockdown of circCOL1A1 significantly increased the apoptosis rate of NPC cells (Fig. 2K). In addition, knockdown of circCOL1A1 promoted the protein expression of cleaved caspase-3 (Fig. 2J). Collectively, these data delineate a clear suppressive effect of circCOL1A1 silencing on the proliferative, invasive, and metabolic phenotypes of NPC cells, underscoring the contribution of circCOL1A1 to the malignant properties of NPC, possibly through the modulation of glycolytic programming. This underpins the Warburg effect as a potential circCOL1A1-driven aspect of NPC pathophysiology.

### CircCOL1A1 competitively binds miR-370-5p

**Fig. 3:**
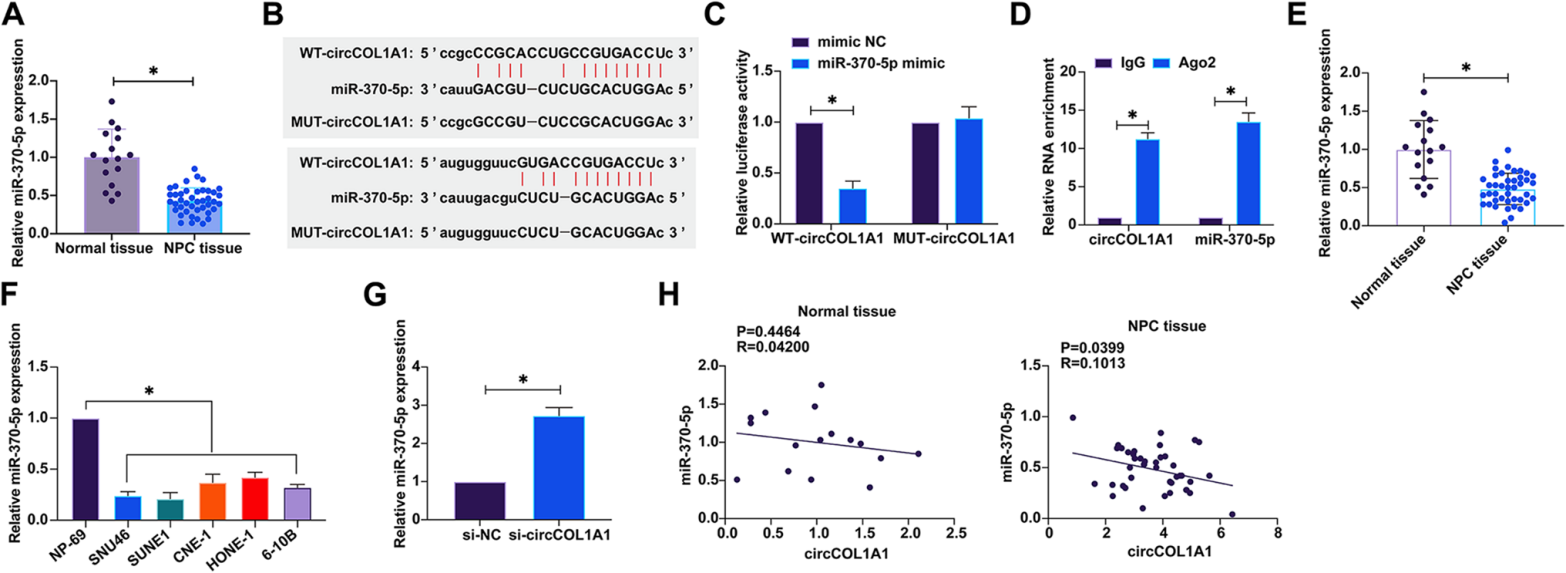
CircCOL1A1 competitively binds to miR-370-5p. **A** The expression of miR-370-5p in NPC tissues and normal tissues was detected by RT-qPCR; **B** Bioinformatics websites predicted potential binding sites between circCOL1A1 and miR-370-5p; **C** Dual-luciferase reporter assay confirmed the target relationship between circCOL1A1 and miR-370-5p; **D** RIP assay confirmed the binding relationship between circCOL1A1 and miR-370-5p; **E** RT-qPCR detected the expression of miR-370-5p in normal tissues and NPC tissues. **F** RT-qPCR detected the expression of miR-370-5p in NPC cell lines and normal cells; **G** RT-qPCR assessed the effect of knocking down circCOL1A1 on the expression of miR-370-5p; **H** Pearson correlation analysis was used to evaluate the correlation between circCOL1A1 and miR-370-5p in NPC tissues and adjacent normal tissues; Data are presented as mean ± standard deviation (n = 3). * P < 0.05. Note: NPC, nasopharyngeal carcinoma; RT-qPCR, Real-time reverse transcriptase-polymerase chain reaction; RIP, RNA immunoprecipitation

To delineate the downstream miRNA landscape modulated by circCOL1A1 in NPC, we employed computational strategies via the starbase platform to predict potential miRNA interactors. From an initial screening of ten miRNA candidates (Supplementary Table S1), seven were previously reported with suppressed expression across diverse cancer types. RT-qPCR analyses revealed that miR-370-5p was underexpressed in NPC tissues compared to adjacent non-cancerous counterparts (Fig. 3A), suggesting that miR-370-5p may act downstream of circCOL1A1. We engineered wild-type circCOL1A1 (WT-circCOL1A1) and mutant circCOL1A1 (MUT-circCOL1A1) constructs with alterations at the predicted miRNA interaction sites (Fig. 3B). Through dual-luciferase reporter assays, we demonstrated that miR-370-5p mimic transfection suppressed the luciferase activity of the reporter harboring WT-circCOL1A1 but not that containing MUT-circCOL1A1 (Fig. 3C), confirming a specific miRNA-target interaction. RNA immunoprecipitation further substantiated the association, with AGO2 complexes preferentially enriching both miR-370-5p and circCOL1A1 (Fig. 3D). The regulatory influence of circCOL1A1 on miR-370-5p was then examined. The expression of miR-370-5p in NPC tissues was significantly higher than that in normal tissues (Fig. 3E). NPC cell lines showed a lower baseline expression of miR-370-5p relative to normal human nasopharyngeal epithelial cells (Fig. 3F). Intriguingly, circCOL1A1 knockdown significantly upregulated miR-370-5p expression (Fig. 3G). Pearson correlation analyses across a cohort of NPC and corresponding normal tissues established a robust inverse correlation between circCOL1A1 and miR-370-5p expression levels within NPC tissues, which was less apparent in normal tissues (Fig. 3H). Together, these data not only corroborate the ceRNA hypothesis for circCOL1A1 via its interaction with miR-370-5p but also implicate this axis as a potentially influential factor in NPC pathogenesis, offering novel insights into the post-transcriptional regulatory networks at play in cancer biology.

### Overexpression of miR-370-5p suppresses malignant behavior of NPC

**Fig. 4:**
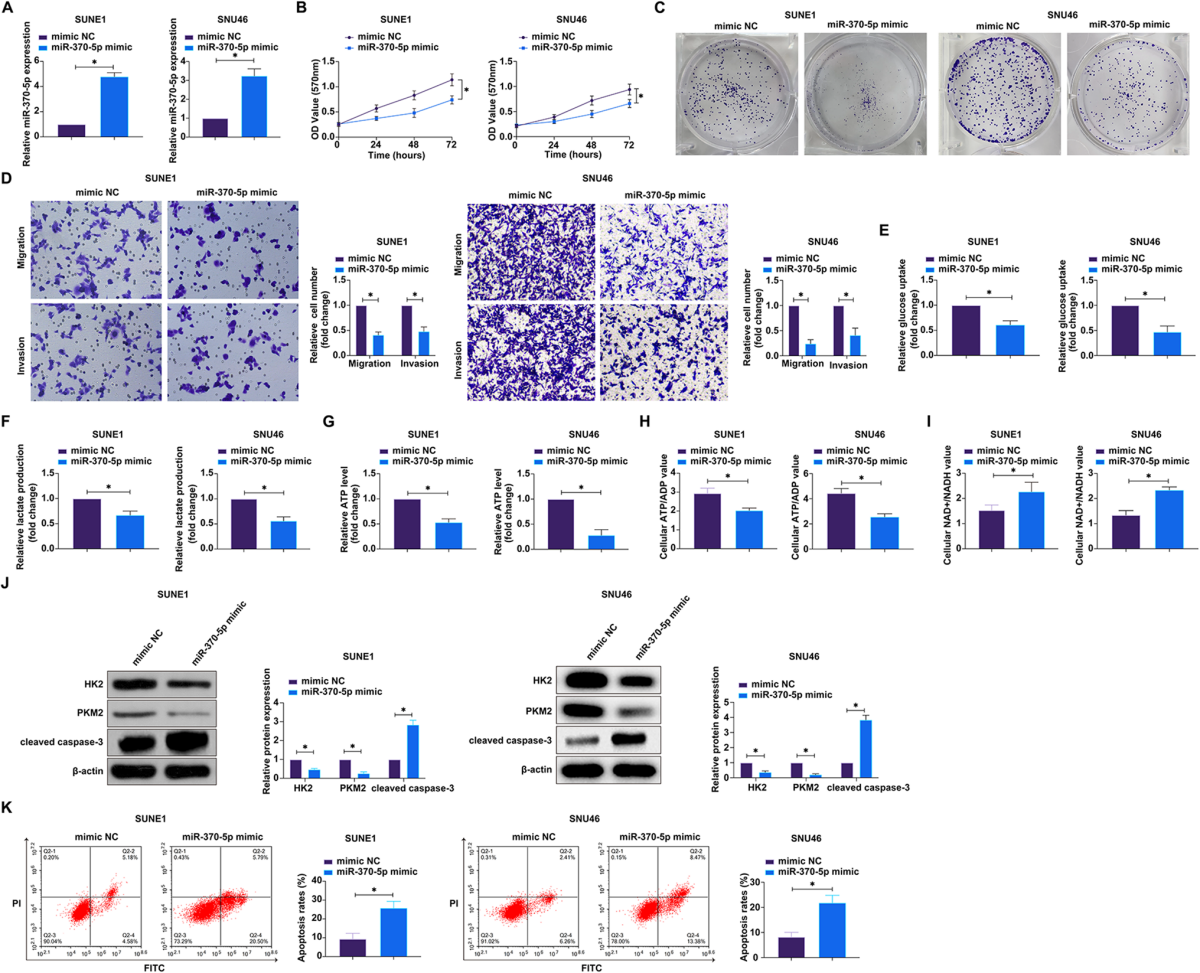
Overexpression of miR-370-5p suppresses the malignant behaviors of NPC. MiR-370-5p mimics were transfected into SUNE1 and SNU46 cells. **A** The expression of miR-370-5p was detected by RT-qPCR; **B** Cell proliferation was measured using the MTT assay; **C** Clonogenic ability was assessed using the plate colony formation assay; **D** Cell invasion and migration capabilities were evaluated by Transwell assay; **E**–**I** Cell glucose consumption, lactate production, ATP levels, ATP/ADP ratio, and NAD^+^/NADH ratio were measured using assay kits; **J** The expression of cleaved caspase-3, HK2 and PKM2 proteins was detected by western blot; **K** Apoptosis rate was detected by flow cytometry. Data are presented as mean ± standard deviation (n = 3). * *P* < 0.05. *NPC* nasopharyngeal carcinoma, *RT-qPCR* Real-time reverse transcriptase-polymerase chain reaction, *MTT* 3-(4,5-dimethylthiazol-2-yl)-2,5-diphenyltetrazolium bromide, *ATP/ADP ratio* adenosine triphosphate/adenosine diphosphate ratio, *NAD*^*+*^*/NADH* Nicotinamide adenine dinucleotide^+^/Nicotinamide adenine dinucleotide, *HK2* hexokinase 2, *PKM2* pyruvate kinases type M2

In light of miR-370-5p's established tumor-suppressive role in colorectal and lung cancers [28, 29], we posited that it might manifest a similar function in NPC. To assess this, miR-370-5p levels were augmented in SUNE1 and SNU46 cells through transfection with miR-370-5p mimic (Fig. 4A). Subsequent assays indicated that miR-370-5p overexpression significantly suppressed cellular proliferation (Fig. 4B), colony formation capability (Fig. 4C), and the invasive and migratory potential of the cells (Fig. 4D). Metabolic profiling revealed a decrease in glucose uptake (Fig. 4E) and lactate secretion (Fig. 4F), alongside a marked reduction in intracellular ATP concentrations (Fig. 4G) and the ATP/ADP ratio (Fig. 4H). Moreover, there was a discernible downregulation in the expression of glycolytic enzymes HK2 and PKM2 (Fig. 4J), concurrent with an elevated NAD^+^/NADH ratio (Fig. 4I). Flow cytometry showed that overexpression of miR-370-5p significantly promoted the apoptosis rate of NPC cells (Fig. 4K) and promoted the protein expression of cleaved caspase-3 (Fig. 4J). These data collectively establish that miR-370-5p overexpression exerts a potent inhibitory effect on the proliferation, invasive capacity, and metabolic reprogramming associated with the Warburg effect in NPC cells.

### The suppressive effect of knockdown of circCOL1A1 on malignant behavior of NPC is partially prevented by knockdown of miR-370-5p

**Fig. 5:**
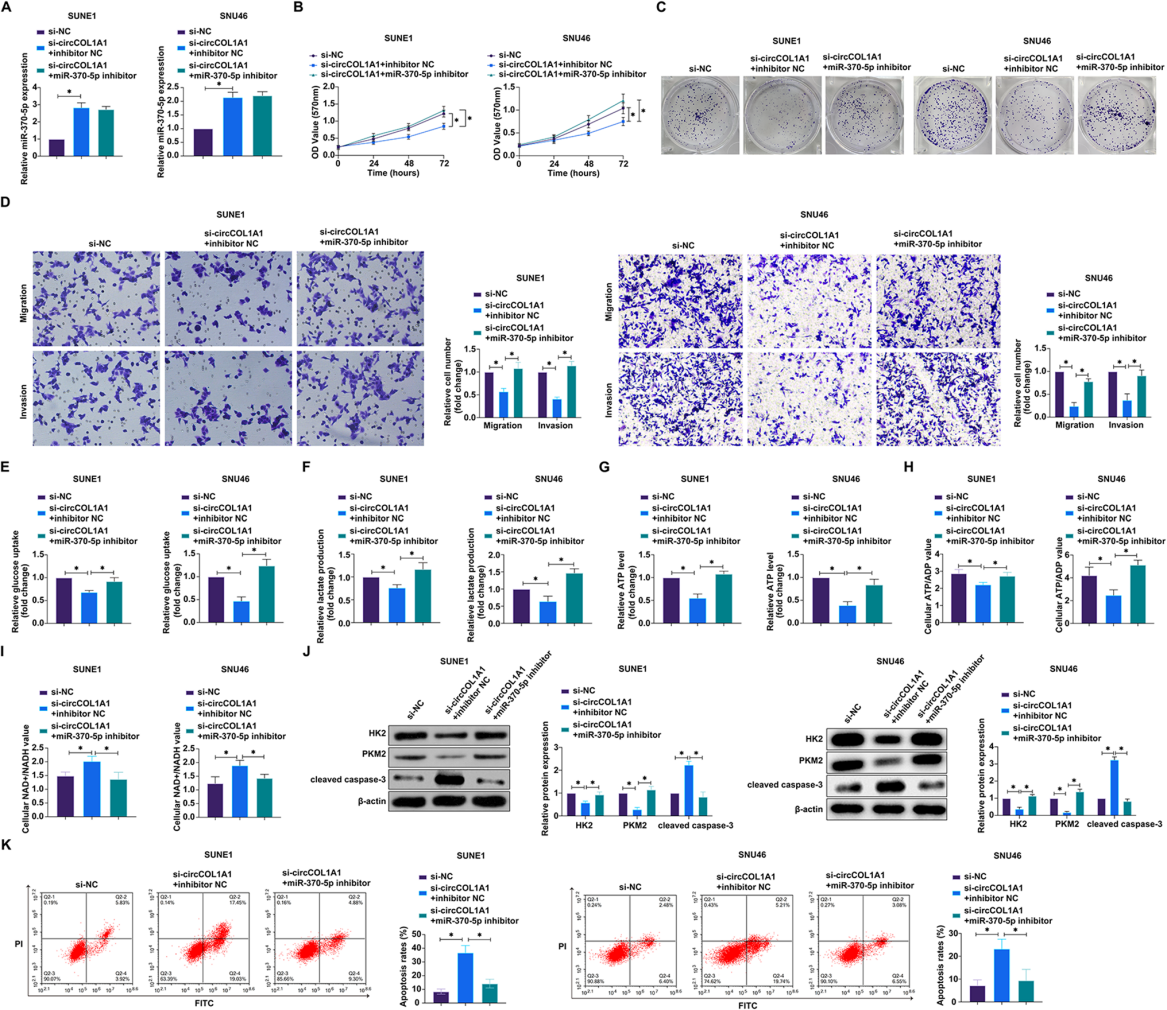
Regulatory effects of miR-370-5p on cell proliferation, invasion, migration, and the warburg phenomenon in NPC. In an experimental setup where SUNE1 and SNU46 cells were simultaneously transfected with si (small interfering RNA)-circCOL1A1 and an miR-370-5p inhibitor, several key cellular and molecular endpoints were assessed. **A** The quantification of miR-370-5p was performed through RT-qPCR analysis; **B** The proliferative capacity of the cells was evaluated using the MTT assay; **C** Clonogenic potential was gauged via plate colony formation assays; **D** The invasive and migratory properties of the cells were investigated using Transwell assays; **E**–**I** Metabolic parameters, including glucose utilization, lactate secretion, ATP concentration, and the ratios of ATP/ADP and NAD^+^/NADH, were determined with specific biochemical kits; **J** Expression levels of the glycolytic enzymes cleaved caspase-3, HK2 and PKM2 were examined by western blot analysis; **K** Apoptosis rate was detected by flow cytometry. The data are represented as the mean ± standard deviation from three independent experiments (n = 3), with * *P* < 0.05 indicating statistical significance. *NPC* nasopharyngeal carcinoma, *RT-qPCR* Real-time reverse transcriptase-polymerase chain reaction, *MTT* 3-(4,5-dimethylthiazol-2-yl)-2,5-diphenyltetrazolium bromide, *ATP/ADP ratio* adenosine triphosphate/adenosine diphosphate ratio, *NAD*^*+*^*/NADH* Nicotinamide adenine dinucleotide^+^/Nicotinamide adenine dinucleotide, *HK2* hexokinase 2, *PKM2* pyruvate kinases type M2

In pursuit of a mechanistic understanding of the role circCOL1A1 plays in governing the malignant behavior of NPC, we delved into the regulatory interplay between circCOL1A1 and miR-370-5p. Utilizing a co-transfection approach, SUNE1 and SNU46 cells were subjected to simultaneous treatment with siRNA targeting circCOL1A1 (si-circCOL1A1) and an antagonist of miR-370-5p (miR-370-5p inhibitor). The RT-qPCR assays revealed that the si-circCOL1A1-mediated enhancement of miR-370-5p expression was effectively negated upon the introduction of the miR-370-5p inhibitor (Fig. 5A). Functional analyses post-transfection presented a compelling narrative: si-circCOL1A1 impeded cellular proliferation (Fig. 5B), diminished clonogenic potential (Fig. 5C), and attenuated the invasive and migratory capacities (Fig. 5D) of the cells. Furthermore, a metabolic shift was evidenced by decreased glucose consumption (Fig. 5E), lactate production (Fig. 5F), ATP levels (Fig. 5G), and a lower ATP/ADP ratio (Fig. 5H), concomitant with an elevated NAD^+^/NADH ratio (Fig. 5I). The si-circCOL1A1 also led to a downregulation of the glycolytic enzymes HK2 and PKM2 (Fig. 5J), yet these molecular and phenotypic alterations were reversed upon the co-administration of the miR-370-5p inhibitor. In addition, the promotion of apoptosis rate and cleaved caspase-3 protein expression by knockdown of circCOL1A1 was reversed by miR-370-5p inhibitor (Fig. 5K, J). These data compellingly suggest that circCOL1A1 modulates the oncogenic attributes of NPC, such as proliferation, invasiveness, migratory propensity, and the metabolic reprogramming reminiscent of the Warburg effect, through the downstream regulatory molecule miR-370-5p.

### miR-370-5p modulates PTMA expression in a targeting manner

**Fig. 6:**
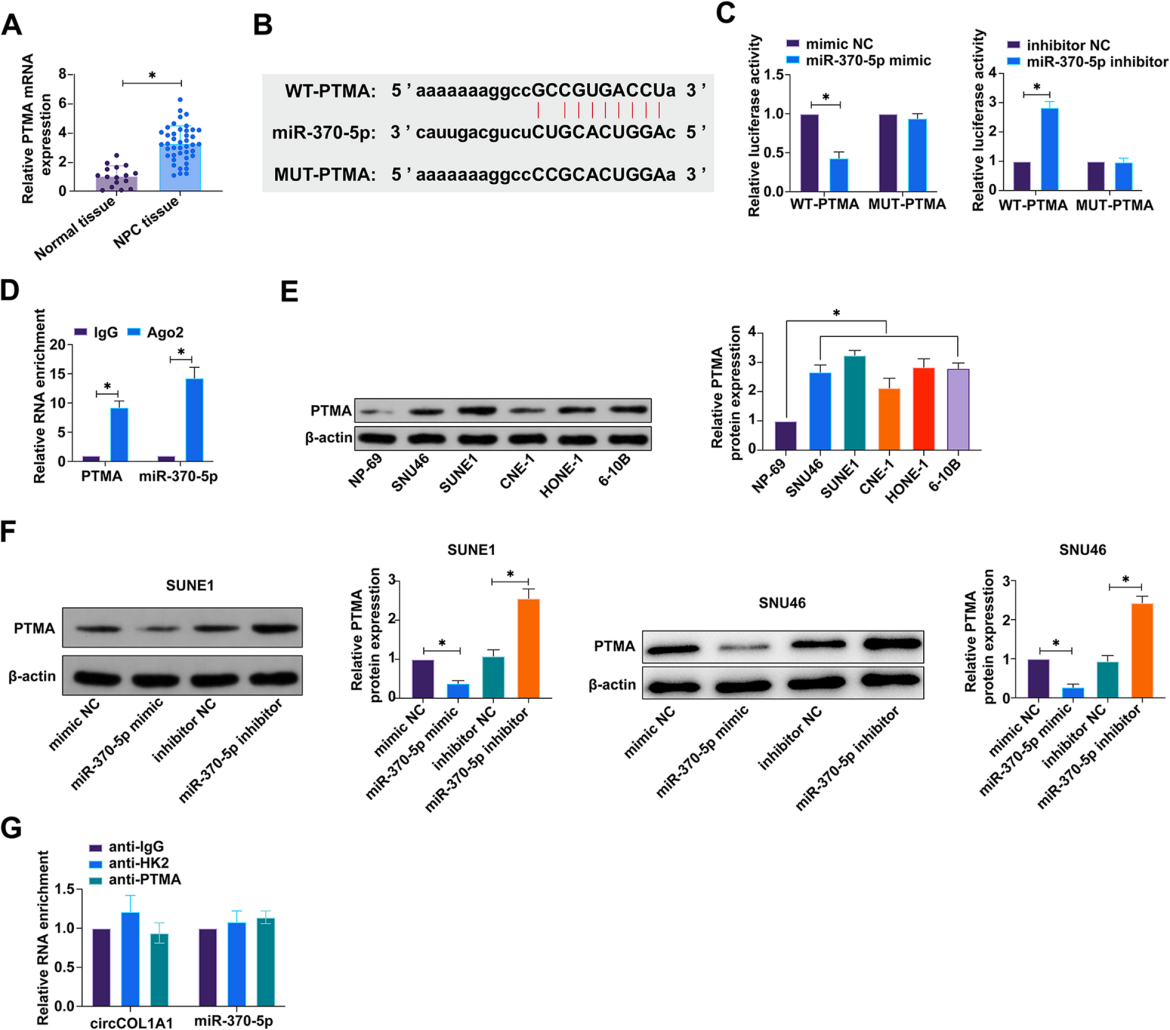
PTMA as a target of miR-370-5p in NPC. **A** The expression profile of PTMA in NPC tissues versus normal adjacent tissues was quantitatively analyzed via RT-qPCR; **B** Bioinformatic predictions highlighted the miR-370-5p binding sequences within PTMA; **C** The targeting relationship between PTMA and miR-370-5p was demonstrated through a dual-luciferase reporter assay; **D** RIP confirmed the molecular association between PTMA and miR-370-5p; **E** Western blot analysis was utilized to compare PTMA protein levels in NPC cell lines with those in normal cell counterparts; **F** The impact of miR-370-5p manipulation on PTMA expression was elucidated through western blot, post knockdown or overexpression treatments; **G** RIP assay to detect direct interaction of circCOL1A1 and miR-370-5p with HK2 and PKM2. Data are summarized as the mean ± standard deviation (n = 3), with * *P* < 0.05 indicating statistical significance. *PTMA*, prothymosin alpha, *NPC* nasopharyngeal carcinoma, *RT-qPCR* Real-time reverse transcriptase-polymerase chain reaction, *RIP* RNA immunoprecipitation

Subsequent to our inquiry into the circCOL1A1-mediated regulatory landscape within NPC, we extended our exploration to potential target mRNAs of miR-370-5p using the bioinformatics platform https://starbase.sysu.edu.cn. An initial screen yielded ten mRNAs as putative targets, with three among them previously documented to exhibit aberrantly elevated expression across diverse cancer types (refer to Supplementary Table S2). RT-qPCR analyses discerned a downregulated expression of PTMA in NPC tissues relative to adjacent non-tumorous tissues (Fig. 6A). Building on the predictive binding sites between PTMA and miR-370-5p, we constructed wild-type PTMA (WT-PTMA) and its mutant counterpart (MUT-PTMA) (Fig. 6B). Dual-luciferase reporter assays demonstrated that co-transfection with WT-PTMA and miR-370-5p mimic significantly attenuated luciferase activity, an effect not mirrored by MUT-PTMA (Fig. 6C). Further, RIP assays indicated a substantial enrichment of PTMA and miR-370-5p within Ago2 complexes (Fig. 6D). Contrasts in expression were also noted in NPC cells, wherein PTMA levels were conspicuously lower as compared to their normal cellular counterparts (Fig. 6E). Subsequent investigations into the modulatory role of miR-370-5p on PTMA expression revealed that knockdown or overexpression of miR-370-5p inversely modulated PTMA protein levels (Fig. 6F). To determine whether circCOL1A1 and miR-370-5p could target and regulate HK2 and PKM2, RIP experiments were performed. As shown in Fig. 6G, neither circCOL1A1 nor miR-370-5p was found to interact with HK2 and PKM2. These findings collectively nominate PTMA as a bona fide target of miR-370-5p, illuminating a hitherto uncharacterized facet of the miRNA's regulatory function within NPC pathobiology.

### CircCOL1A1 by miR-370-5p/PTMA axis promotes NPC malignant behaviors

**Fig. 7:**
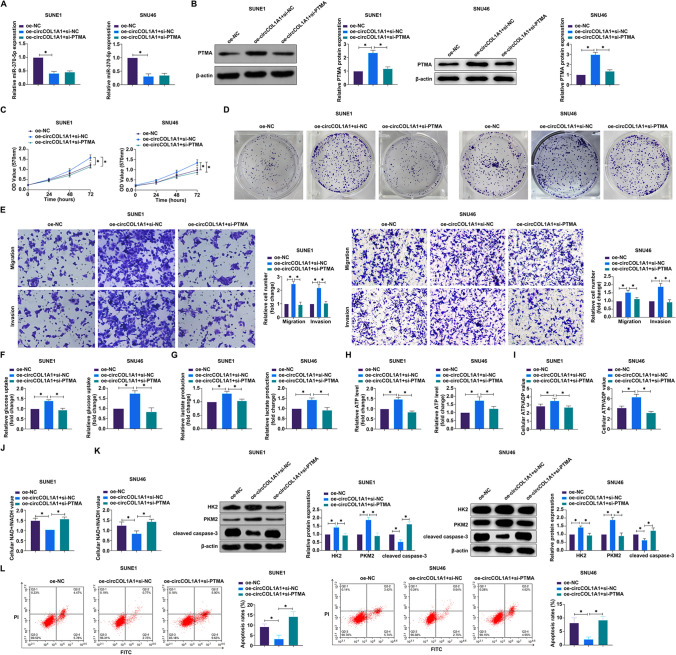
CircCOL1A1 promotes malignant behaviors in NPC via the miR-370-5p/PTMA axis. In an experimental model where SUNE1 and SNU46 cells were co-transfected with oe-circCOL1A1 (overexpression plasmid for circCOL1A1) and si-PTMA (PTMA silencing RNA), a series of assays were conducted. **A** The expression levels of miR-370-5p were quantified using RT-qPCR; **B** Western blot analysis was performed to assess the expression of PTMA protein; **C** Cell proliferation was evaluated using the MTT assay; **D** The capability for colony formation was determined using a plate colony formation assay; **E** The invasive and migratory potential of the cells was measured by Transwell assays; **F**–**J** Metabolic activity, including glucose consumption, lactate production, ATP concentration, and the ratios of ATP/ADP and NAD^+^/NADH, were analyzed using respective assay kits; **K** The expression of metabolic enzymes cleaved caspase-3, HK2 and PKM2 was probed by western blot; **L**: Apoptosis rate detected by flow cytometry. The presented data reflect the mean ± standard deviation from three independent experiments (n = 3), with * *P* < 0.05 signifying statistical significance. *PTMA* prothymosin alpha, *NPC* nasopharyngeal carcinoma, *RT-qPCR* Real-time reverse transcriptase-polymerase chain reaction, *MTT* 3-(4,5-dimethylthiazol-2-yl)-2,5-diphenyltetrazolium bromide, *ATP/ADP ratio* adenosine triphosphate/adenosine diphosphate ratio, *NAD + /NADH* Nicotinamide adenine dinucleotide^+^/Nicotinamide adenine dinucleotide, *HK2* hexokinase 2, *PKM2* pyruvate kinases type M2

To interrogate the involvement of the miR-370-5p/PTMA regulatory axis in circCOL1A1-mediated oncogenic pathways in NPC, we engineered SUNE1 and SNU46 cells with an overexpression construct for circCOL1A1 (oe-circCOL1A1) alongside siRNA targeting PTMA (si-PTMA). Analysis demonstrated that oe-circCOL1A1 diminished miR-370-5p expression while concurrently augmenting PTMA levels, a regulatory effect that was nullified upon introduction of si-PTMA (Fig. 7A, B). Perturbation experiments revealed that oe-circCOL1A1 precipitated a marked increase in SUNE1 and SNU46 cell proliferation rate (Fig. 7C), clonogenicity (Fig. 7D), invasiveness, and migratory behavior (Fig. 7E), together with enhanced glucose uptake (Fig. 7F), lactate production (Fig. 7G), ATP concentration (Fig. 7H), and ATP/ADP ratio (Fig. 7I). This was paralleled by an elevated expression of the metabolic enzymes HK2 and PKM2 (Fig. 7K), with a concomitant decrease in the NAD^+^/NADH ratio (Fig. 7J). Flow cytometry showed that the inhibitory effect of overexpression of circCOL1A1 on apoptosis rate and cleaved caspase-3 was reversed by knockdown of PTMA (Fig. 7L, K). Notably, these pro-tumorigenic effects elicited by oe-circCOL1A1 were effectively counteracted by the si-PTMA co-transfection. Collectively, our data delineate a novel mechanistic insight, demonstrating that circCOL1A1 potentiates NPC tumorigenesis and metabolic remodeling via modulation of the miR-370-5p/PTMA axis, underpinning a potential therapeutic target within the oncogenic circuitry of NPC.

## Discussion

Local recurrence and distant metastasis of nasopharyngeal carcinoma hinder the clinical therapeutic effect of nasopharyngeal carcinoma, so it is crucial to explore its mechanism and find the active molecules in the mechanism. Recent studies have shown that miRNAs [30] and circRNAs [20] are in essence in NPC, such as miR-506 [31] and circ_0000215 [32]. Here, circCOL1A1 was determined to regulate the miR-370-5p/PTMA axis to produce oncogenic effects in NPC. Therefore, it can serve as a new model for studying circRNA regulatory mechanisms and developing tumor drug targets.

circCOL1A1 was only selected because it is highly abundant and stable in NPC cells. Furthermore, it was more rich in NPC tumor tissues and was associated with lymph node metastasis and TNM staging. Follow-up analysis of cases is requested in future studies to clarify whether circCOL1A1 is able to be an effective marker for liquid biopsy. In NPC, some circRNAs have been reported to be oncogenes that promote NPC development [21]. A previous paper analyzes that circCOL1A1 is involved in the malignant behavior of cancer cells [23]. Similarly, the current work revealed that circCOL1A1 downregulation blocked NPC cell malignancy, suggesting that circCOL1A1 may have an oncogenic function during NPC progression.

It is now recognized that even with adequate oxygen, cancer cells tend to meet their energy needs through aerobic glycolysis rather than oxidative phosphorylation, thereby promoting cancer cell proliferation and growth and ultimately promoting the malignant development of cancer [33–35]. A large amount of lactate produced by glycolysis changes the microenvironment of tumor cells, inducing tumor cell invasion and metastasis [36] whereas inhibiting aerobic glycolysis reduces the proliferation and invasive capacity of NPC cells [37]. The work revealed a critical role for circCOL1A1 in NPC progression, but previous studies have provided little evidence for the function of circCOL1A1 in cancer metabolism. Interestingly, data analysis in this study showed that silencing circCOL1A1 decreased cellular glucose consumption, lactate production, ATP levels and ATP/ADP and increased NAD^+^/NADH, as well as decreased glycolysis-related proteins HK2 and PKM2. Intriguingly, circCOL1A1 is posited to modulate the metabolic landscape of cancer cells through a miRNA sponging mechanism, particularly by sequestering a cohort of miRNAs including miR-370-5p, thereby attenuating their suppressive impact on multiple downstream target genes. These targets likely encompass key glycolytic enzymes such as HK2 and PKM2, whose hyperactivation is directly implicated in the Warburg effect [38, 39]. Knockdown of circCOL1A1 resulted in the release of miR-370-5p's repressive hold, consequently reinforcing the inhibition of these enzymes and curtailing the upregulation of glycolysis. Moreover, a reduction in intracellular ATP levels may reflect a metabolic shift from glycolysis to oxidative phosphorylation [40], a transition that potentially involves the activation of AMP-activated protein kinase (AMPK) [41], a sentinel in cellular energy homeostasis under conditions of energy deprivation. Activation of AMPK is known to curb the growth and proliferation of tumor cells and propel a reprogramming of cellular energy metabolism [41]. The observed depletion of ATP levels intimates that cells may be transitioning away from glycolysis as the principal energetic pathway, instead favoring aerobic metabolism [42]. A direct repercussion of dampened glycolytic throughput could be an elevated NAD^+^/NADH ratio, given that NAD^+^ is more efficiently recycled during aerobic respiration [43].

To explore the regulatory mechanism of circCOL1A1 in NPC, the subcellular localization of circCOL1A1 was analyzed and it was mainly localized in the cytoplasm of NPC cells, suggesting that circCOL1A1 mainly acts as a ceRNA of downstream miRNAs (miR-370-5p was selected) to play a post-transcriptional regulation role on downstream gene expression. Interestingly, miR-370-5p expression was lower in NPC tissues and cells. Previously, miRNAs have been shown to be important regulators of cancer progression and metabolic reprogramming [44, 45]. MiR-370-5p confers tumor-suppressing actions in some human cancers and has the potential to suppress the malignant phenotype of cancer cells, such as breast, lung [28], and colorectal cancer [29]. Consistent with previous studies, our data proved that miR-370-5p had the potential to suppress the malignant phenotypes of NPC cells.

It is well known that miRNAs usually bind to the 3'UTR end of downstream target genes [46]. As a target gene of miR-370-5p in this work, PTMA, a nuclear protein, has been reported to be activated in esophageal cancer [47], colorectal cancer [48], and glioma [49]. Importantly, a recent study shows that PTMA is downregulated in NPC and involved in miR-1-induced apoptosis [50]. Consistent with these findings, PTMA was expressed lowly in NPC.

Although this study found that circCOL1A1 promotes NPC progression by miR-370-5p/PTMA axis, it has not been further validated in vivo. In addition, circCOL1A1 expression in the serum of NPC patients should be detected in the follow-up analysis to verify whether it is a biomarker for blood biopsies of NPC patients. Finally, functional and signaling pathways should be expanded to elucidate how aberrant PTMA expression promotes malignant tumor progression through metabolic reprogramming.

## Conclusion

We report the identification of a novel circRNA, circCOL1A1, which has potent oncogenic activity in NPC. Furthermore, we demonstrate that circCOL1A1 upregulates PTMA expression by miR-370-5p, thereby supporting the Warburg effect of NPC cells to promote cancer cell proliferation, migration and invasion. This study extends the understanding of circRNA function in NPC pathogenesis and proposes a novel circRNA as a potential biomarker and therapeutic target for NPC.

### Supplementary Information

Additional file1 (XLSX 11 KB)

Additional file2 (XLSX 11 KB)

## Data Availability

The datasets used and/or analyzed during the present study are available from the corresponding author on reasonable request.
